# Evaluation of Cueing Innovation for Pressure Ulcer Prevention Using Staff Focus Groups

**DOI:** 10.3390/healthcare2030299

**Published:** 2014-07-25

**Authors:** Tracey L. Yap, Susan Kennerly, Kirsten Corazzini, Kristie Porter, Mark Toles, Ruth A. Anderson

**Affiliations:** 1 School of Nursing, Duke University, Durham, NC 27710, USA; E-Mails: kirsten.corazzini@duke.edu (K.C.); ruth.anderson@duke.edu (R.A.A.); 2 Center for the Study of Aging and Human Development, Duke University, Durham, NC 27710, USA; 3 School of Nursing, University of North Carolina at Charlotte, Charlotte, NC 28223, USA; E-Mail: skenner2@uncc.edu; 4 RTI International, Research Triangle Park, NC 27709, USA; E-Mail: porter@rti.org; 5 School of Nursing, University of North Carolina at Chapel Hill, Chapel Hill, NC 27599, USA; E-Mail: mtoles@email.unc.edu

**Keywords:** pressure ulcer prevention, long-term care, multidisciplinary healthcare teams, nursing interventions, diffusion of innovation, implementation

## Abstract

The purpose of the manuscript is to describe long-term care (LTC) staff perceptions of a music cueing intervention designed to improve staff integration of pressure ulcer (PrU) prevention guidelines regarding consistent and regular movement of LTC residents a minimum of every two hours. The Diffusion of Innovation (DOI) model guided staff interviews about their perceptions of the intervention’s characteristics, outcomes, and sustainability. Methods: This was a qualitative, observational study of staff perceptions of the PrU prevention intervention conducted in Midwestern U.S. LTC facilities (*N* = 45 staff members). One focus group was held in each of eight intervention facilities using a semi-structured interview protocol. Transcripts were analyzed using thematic content analysis, and summaries for each category were compared across groups. Results: The *a priori* codes (observability, trialability, compatibility, relative advantage and complexity) described the innovation characteristics, and the sixth code, sustainability, was identified in the data. Within each code, two themes emerged as a positive or negative response regarding characteristics of the innovation. Moreover, within the sustainability code, a third theme emerged that was labeled “brainstormed ideas”, focusing on strategies for improving the innovation. Implications: Cueing LTC staff using music offers a sustainable potential to improve PrU prevention practices, to increase resident movement, which can subsequently lead to a reduction in PrUs.

## 1. Introduction

Reducing pressure ulcers (PrU) has proven difficult for U.S. long-term care (LTC) facilities [1,2]. PrUs are areas of soft-tissue injury that occur when there is compression involving a bony prominence and an external surface [3]; they are painful and associated with septic infections and premature deaths [4]. LTC is the fastest growing segment of the U.S. healthcare continuum, and LTC residents have a higher prevalence of risk factors for PrU development than community-dwellers; LTC residence itself is a risk factor [5]. Many PrUs are avoidable [6], and treatment cost is greater than prevention cost, provoking a quest among stakeholders to balance cost containment, quality, and care delivery to prevent facility-acquired PrUs. The cornerstone of ulcer prevention is eliminating the source of pressure, usually accomplished through some form of movement [7]. In a recent randomized intervention trial [8], we showed that odds of acquiring a new PrU were lower in facilities that used tailored music cues, played every two hours during the 12-h daytime period to remind the multidisciplinary LTC staff [9] to encourage or enable all residents to move, regardless of the residents’ apparent risk for PrU. Given the clinical significance of this finding, we aimed to understand what factors contributed to the staffs’ implementation and adoption of the intervention and observed reduction in PrUs. We used the Diffusion of Innovation (DOI) model to frame questions for staff focus groups [10]. The DOI model is considered useful for exploring the characteristics of any idea, device, or method perceived as new by an individual or group, in this case, using musical cues to prompt the movement of LTC residents as a PrU prevention strategy. Our research question was: in what ways did the staff describe the innovation to be sustainable and either better or worse than previous methods? The DOI identifies five characteristics that influence an individual’s decision to adopt or reject an innovation: observability (overall visibility); trialability (how easily it can be experimented with); compatibility (for assimilation into routine); relative advantage (over a previous method) and perceived complexity [10]. Because the diffusion of innovations involves a process by which communication flows through specific channels over time among the members within the user system, the human perception of the innovation and its diffusion and impact on outcomes is important to the innovation’s successful implementation. Therefore, all staff in eight facilities that received the intervention (either for 6 or 12 months), were asked to share their perceptions of the intervention’s attributes, impact and sustainability.

## 2. Experimental

### 2.1. Design, Sample and Setting

This qualitative study of LTC staff perceptions of a musical cue innovation used a naturalistic inquiry method focus group design, whereby multiple focus groups are conducted with facility staff. Focus groups were chosen, because they allow for ideas and perspectives to emerge from the interactions that might not occur with individual interviews [11]. The Institutional Review Board approved all study procedures, and all participants provided written informed consent for study participation. Administrators in the facilities limited us to one focus group per facility.

Within 2 weeks of completing the intervention trial, focus group members were recruited in each of the eight facilities, using a purposive sampling design [11] to enable a comparison across facilities. Participants from all departments were recruited using flyers containing the purpose and logistics about the study. We asked the director of nursing or administrator to distribute the flyers to all staff in each LTC facility. Any staff member who had participated in the intervention trial for at least two months was eligible. Interested participants contacted the study personnel by phone if they were interested in participating on the scheduled date for the onsite focus group; snacks and drinks were served. A total of 45 participants across the eight facilities volunteered and participated, because they had compatible shifts and workloads; some staff participated off-clock (either coming in early or staying just past their shift completion).

Two PhD-level study team members experienced in focus group methods moderated each focus group session. Each session was completed within a 1-h time frame. The moderator provided an overview of the study’s purpose, background and procedures, such as investigator roles and session recordings, which was followed by introductions. Moderators encouraged all group members to participate in the discussion and explored dissenting views. All eight focus groups were audio-recorded and transcribed verbatim. The PI listened to the recording and verified the accuracy of transcripts before loading into the qualitative analysis program, NVivo 9.0 [12], for analysis.

### 2.2. Focus Group Interview

The team developed a semi-structured interview guide designed to elicit participants’ descriptions of their experiences with the intervention, opinions and descriptions of the innovation and its implementation and perceived outcomes. The questions (Table 1) were written using six *a priori* codes (observability, trialability, compatibility, relative advantage, complexity and sustainability) to elicit information regarding barriers and facilitators and participants’ feelings and expectations about the intervention. We developed *a priori* codes for the DOI model’s five innovation characteristics to explore the degree to which each attribute affected adoption, and the sixth code, sustainability, to understand what contributes to the long-term endurance of the innovation.

**Table 1:** Questions, definitions and representative quotations.

Questions	Innovation Characteristics: *A Priori Codes*	Themes	# of quotes	% of Quotes	% of Conversation	# Groups Who Discussed	Exemplar Quotation(s) from Focus Groups
	**Observability **				**21%**	**8**	
What have you seen happen as a result of this program?	(*discern benefits from innovation*)	*Results visible*	65	16%			“It just helps the residents, I mean they will dance with us, and they usually didn’t do that. I think they enjoyed… they enjoyed the music a lot.”
		*Results not visible*	10	3%			[Music was] “Kind of a side benefit, I would conjecture from the actual looking at the skin issues, probably held about the same, I would conjecture …. So you don’t know that there is any difference necessarily, then? I would guess probably not.”
	**Trialability **				**12%**	**7**	
What were your expectations of the pressure ulcer prevention program? Were these expectations met or not? Please describe.	(*can experiment with or adapt innovation*)	*Can experiment/adapt*	45	11%			“first management team picked music and didn’t do that again … then half staff, half resident music picks”
		*Cannot experiment/adapt*	4	1%			“some departments did not get involved”
	**Compatibility **				**38%**	**8**	
How did you feel about participating in the pressure ulcer prevention program?	(*users’ values, norms, and needs are met and fits workflow*)	*Good fit*	74	19%			“it really wasn’t too far from our routine, it was just a different way to approach it” “very good that it was interdisciplinary … It’s an opportunity for everybody”
		*Poor fit*	72	18%			“double the paperwork” “[Sundays] we usually have a guest reading scripture and then the music would play. They usually get here between 10:00 and 12:00 and that’s when the music plays … and you got “Johnny B Goode” coming on and he’s trying to preach”
	**Relative Advantage**				**8%**	**8**	
What have you seen happen as a result of this program?	(*usual care* vs*. previous standard of care approach*)	*Perceived as better; adoptable*	37	9%			“it has been more of a quality of life thing, than a clinical thing”
		*Perceived as worse; not adoptable*	0				no examples
	**Complexity**				**7%**	**7**	
Were there any barriers to your ability to perform the activities requested when the musical prompt occurred?	(*Intrinsically complex; Level of degree of use*)	*Perceived as simple*	13	3%			“good reminder for both staff and residents”
		*Perceived as difficult*	18	5%			“you are saddled with even more detailed record keeping” “Dietary, if it was during lunch, it’s just that they are busy”
	**Sustainability**				**9%**	**8**	
What is there about this pressure ulcer prevention program that you can still use once this program ends?	(*extent/degree innovation is used over time*)	*High degree of support*	15	4%			“everyone got into a routine and went with it”
		*Low degree of support*	20	5%			“I think overall it started well, but kind of slacked with staff as the program went on. Probably due to lack of enforcement, you know, on all of our parts.”
		*Brainstormed Ideas* (ways to improve the system for successful future adoption and implementation of the innovation)	25	**6%**	**5%**		“touchscreen for documentation” “we realized half way through the study we should have had a blog site for all the facilities involved”

The intervention to which the questions referred has been previously described [8]. Briefly, music served as an auditory cue (timely reminder) to staff that all LTC residents should be moved or reminded to move. The pre-existing organizational standard of care had been to reposition only at-risk residents at least every 2 h, per commonly accepted PrU preventive practices [13]. Each facility established mobility teams, which were nurse-led and included representatives from nursing, administration, dietary, housekeeping, activities coordinator, maintenance, physical therapy, chaplaincy, and residents’ visiting family members. Nursing staff repositioned residents who required assistance, while ancillary staff reminded mobile residents to change positions or walk. Musical selections played facility-wide every 2 h during 12 daytime hours, 7 days a week. Every month, staff and residents at each facility selected 10 new songs (with or without lyrics). A laptop computer with standard task-manager software was configured to select 6 of the 10 full-length audio music files at random each day to be played over the facility’s hallway PA system. Resident movement was recorded on a study-specific document. When the study launched, a whiteboard was meant to be used for visual documentation to determine which residents had been missed, but every facility disliked the boards, and they were not used.

### 2.3. Analysis

Because we used the DOI theory to guide the qualitative content analysis [14], we used a directed approach to identify core concepts in the focus group data. We derived our *a priori* codes from the theory [14]. Brief definitions for the DOI *a priori* codes are presented in Table 1. The analytic plan allowed for new themes to emerge *post hoc*.

**Figure 1:**
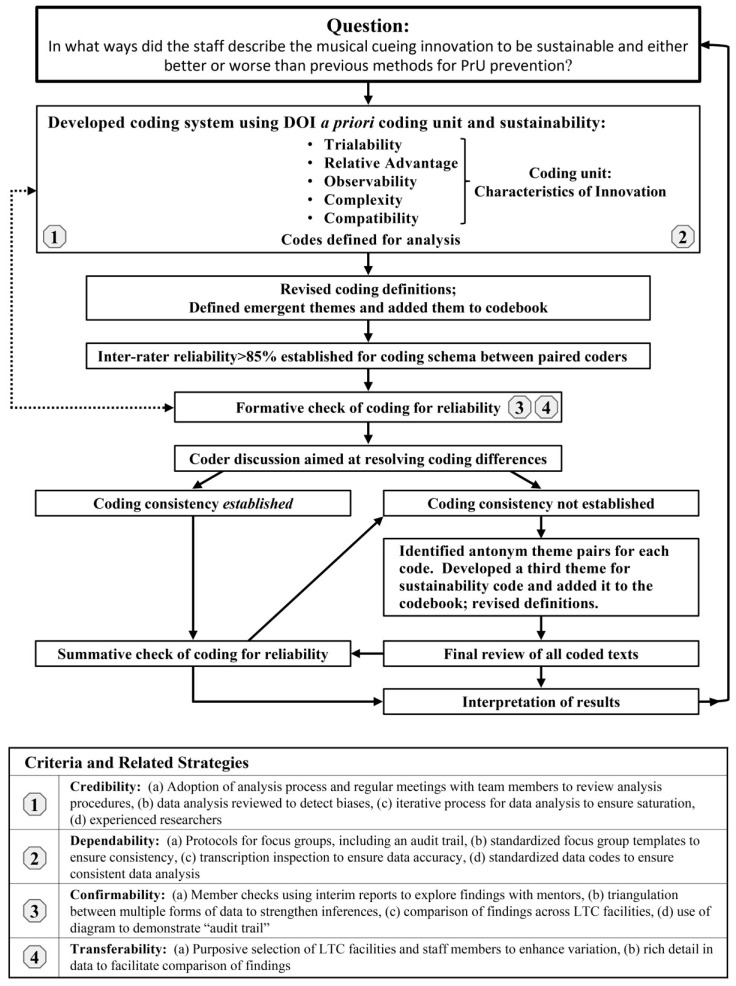
System of analysis. PrU, pressure ulcer; DOI, Diffusion of Innovation.

Four criteria to ensure analytical rigor were employed in the development of the analysis procedures described below (Figure 1) [15,16]. The study should measure what it was intended to (credibility), and if the work was repeated in the same way, in the same context and with the same participants and methods, one should get similar results (dependability). The findings should be the results of the experiences of the participants (confirmability) and should be applicable to other situations (transferability).

The interdisciplinary focus group analysis team included six investigators with backgrounds in gerontological nursing, social gerontology, public health, education, and organizational science. To establish consistency among the six coders, all team members coded the same data excerpts from one transcript, discussed the coding decisions, and refined code definitions. Next, each coder independently completed coding the rest of the relevant text of the transcript, and the group of coders met to re-inspect all codes. A codebook with definitions was developed to increase trustworthiness (Figure 1).

Next, a pair of coders was assigned to each transcript and coded them independently using the established definitions for codes and themes. The coder pairs met to examine the coding that each had applied to the text and to compare the coding with each other to establish reliability. The pairs of coders established inter-coder reliability at above 85% [17]. To increase the trustworthiness, the full coding team met repeatedly, until the full group had reviewed all coded statements for the consistency and accuracy of codes. Differences in coding were discussed and resolved by the team. Data emerged that could not be coded with *a priori* codes or emergent themes and was grouped into a seventh theme (brainstormed ideas). Next, the PI and one other coder met to examine and ensure that all transcripts and coding assignments had been appropriately coded according to correct definitions across group transcripts.

## 3. Results

A total of 45 staff members, 98% female, participated in the study, which included 26 certified nursing assistants (CNA), seven licensed practical nurses (LPN), eight registered nurses (RN), two housekeepers and two dietary staff. Each focus group exceeded the suggested minimum and mean sample size of five to eight participants [11,18]. Theme saturation appeared to be reached after the multiple facility cases (five groups), because no new information was emerging and no disconfirming evidence was found that enhances transferability. There were two types of responses that we noted for each of our codes, one being affirmation or a positive response by participants and the other being a negation or a negative response by participants to the characteristics of the innovation. Table 1 presents each characteristic coded and the subsequent two themes, the frequency and percent of comments related to each theme, the number of focus groups that discussed the category, and examples of those comments. The calculated percentage of the conversation for all groups was decided by tallying the number of discrete quotes (each quote that fit the predetermined definition) for each theme that emerged from the *a priori* category and then dividing the number of discrete quotes within each category by the total number of quotes. No substantial differences were found between the groups’ participation and the comments yielded. The results will be reported below by each *a priori* category for all eight groups together.

### 3.1. Observability

The two themes related to observability of the innovation included: (1) the visible benefits of the innovation relative to staff and resident behavior and the PrU outcomes; and (2) no obvious benefits. The visible benefits included cueing being enjoyable and PrUs decreasing. All groups discussed the music as being a fun cue that resulted in an increase in both movement and quality of life for both staff and residents. One staff commented that the “music was fun…what came out more than anything is how much the residents enjoyed the music”, and another staff member mentioned how the music “improved staff and resident mood”. The staff also believed they were seeing a decrease in the number of facility-acquired PrUs, remarking that “everyone participated; we’ve seen less ulcers”. The theme for the absence of visible benefits of the innovation included comments about the dearth of multidisciplinary involvement and some staff members failing to see the importance of regular movement. In some facilities, the lack of multidisciplinary effort was disappointing to others: “some departments did not get involved as planned”. They also noted that if you did not have many residents who were bed ridden, some staff failed to see the importance of regular resident movement: “so from my standpoint, it was sort of, I just got done with exercise class and now the music’s going off. ‘Okay, everybody, go again, let’s move around a bit”. Overall, 21% of the focus groups’ discussion showed that staff could discern benefits from the innovation. This means that 65 of the 75 discrete quotes in this category were about results that were visible to them. This high level of visible benefits thereby enhances the perception of the innovation’s added value when adopted.

### 3.2. Trialability

The two themes related to the trialability of the innovation included: (1) it can be “tinkered” with and adapted for our facility; and (2) it cannot be experimented with to fit our facility. Several adjustments were made to fit facility requests, such as music choices, cueing times, documentation forms, and the placement of the documentation binder for different disciplines. Not every facility expressed a need for changes, *i.e.*, facilities were blinded to each other’s adjustments during the intervention study. When the adjustments were later discussed across the focus groups, it became apparent that some of the changes may have worked in other intervention facilities. For example, one participant stated, “We gave Housekeeping their own documentation; we gave Dietary their own documentation”. When group moderators asked if this would have been helpful in other facilities, those staff members agreed. Another example of tweaking the innovation was that music was customized several ways, including both the timing and type of music: (1) “we had to adjust a little bit, not always with the music at mealtimes …, but the other times where you could”; and (2),

“when you play music that came from their era, they started with the memories because I know I brought The Platters out to play in the Dining Room, and then when I’d be in the Dining Room, we’d start talking to the husbands, and they would start talking about going dancing with their wives at the Park, and for some of the residents they would start to reminiscing and it was …. The Platters were soft, and their songs are back from when they were younger, they really liked it”!

However, there also were instances in which the innovation was not successfully adapted as planned, with statements like “we didn’t fool with the whiteboard a lot” and “I think the music was a good trigger to remind people, you know, to move your people as long as everybody was participating.” Overall, the intervention was deemed pliable and could be adapted to each facility to enhance adoption potential. Ninety-two percent (45 of 49) of the comments were rated as reflective of the staff’s perceived ability to experiment with or adapt the innovation.

### 3.3. Compatibility

The two themes related to the compatibility of the innovation included: (1) the innovation was well-matched with the staff values, norms, and perceived needs and fits in the current workflow; (2) the innovation was not well-matched and did not fit the current workflow. In the focus group discussions, the topic of compatibility involved 38% of the overall conversation with an even split between the dichotomous themes. The attributes of the innovation that were considered a good match with staff values and needs were the perceived increase in resident participation and movement and the multidisciplinary involvement. One participant commented that, “I think in the beginning, from my perspective, I didn’t feel like it was anything that we didn’t know we should already be doing. I thought it was an excellent opportunity to refresh and reeducate and remind people how important it is to minimize your skin breakdown and your disease from not moving. And, I think that it was very good that it was interdisciplinary, that Housekeeping, everyone was involved because it is an opportunity for everybody to take part in the therapy.” Moreover, a former housekeeper noted, “that’s one of the reasons I became an aide, “cause as a housekeeper you can’t do a whole lot. [Once I started this program I] wanted to have hands-on”. Another staff member described that staff were “surprised at the number of residents when the music would go off; they would say, “there’s the music, we gotta’ move!” However, some components were not perceived to be as well-matched to staff and resident needs. For example, “documentation fell back on aides” in some facilities and was generally considered an unwanted increase in work. Staff in all facilities “backed away from the white board when we realized it was really duplication of a lot of things; maybe we didn’t capture the interdisciplinary teams as well, going away from the board, but we still caught the documentation in the binders that were supplied”. Staff comments about compatibility were almost evenly split between perceptions of a good fit *versus* poor fit for the innovation. When staff considered how well the innovation fit into their already established routine, they saw the innovation as being close to, yet somewhat different from, their accustomed work flow. While staff were willing to consider making changes in routine, the driving force behind perceptions of poor fit were things contributing to an increased workload, such as study-related documentation.

### 3.4. Relative Advantage

The only theme relating to the relative advantage of the innovation was that it was perceived as better than the previous method used to ensure resident movement on a regular basis. The emotional uplifting of the music and the increased interaction with the residents was considered an advantage. All groups described how the music “spunks us up a little bit”, and several described the residents and how “they dance with us, and they don’t usually do that”. There was no discussion alluding to any part of the musical cues being considered as worse than the previous standard of care; however, the additional documentation required for the study was not considered to be advantageous. This intervention was considered an advantage over the previous method used for resident movement to prevent PrUs and was perceived to positively influence their decisions to adopt. Eight percent of the total comments reflect the staff’s perception that usual care is enhanced by supplementation of a cueing system; all comments within this category (37/37) considered this music innovation as better.

### 3.5. Complexity

The two themes related to the complexity of the innovation included: (1) the innovation is perceived as simple to use and not difficult; and (2) the innovation is intrinsically complex and hard to do. Changing of the music and responding to the musical cue were perceived as simple and easy to use. All groups provided examples of how they “were able to change the music” and let it evolve over time to satisfy everyone in the facility. It was also noted that the music “helped residents stay on schedule for other tasks”, as one staff member said:

“My alert residents mentioned that [the music] helped them keep on schedule if they had therapy or lunch, or know what to do because they got to that 2 h mark, and they would be like, ‘Oh, the music is always time to go to an activity, or it’s time… [for smoke break]’ so they actually started timing their schedule and their days around what was going on with the music, so that was something I know wasn’t part of the program, but it helped them to know what time it was, and that they needed to get to where they were going”.

A lack of innovation ease was perceived when the music cue was not appropriate for certain time periods, for example, “when passing trays it was a timing issue”. All participants noted that in some locations, it was harder to hear the music, for example: “I’m in the Rehabilitation Department, and we couldn’t really hear the music back there”. All groups also mentioned difficulties in accomplishing the added documentation related to resident movement. Overall, the decision to adopt was affected by both the perceived complexity and perceived simplicity of the innovation; however, only 3% of the conversation across groups was about the innovation simplicity and 5% was about the perceived intrinsically complex components.

### 3.6. Sustainability

Three themes emerged related to the sustainability potential and subsequent adoption of the innovation: (1) the innovation continues to be used over time; (2) the innovation does not continue to be used over time; and (3) brainstormed ideas, defined as staff ideas or proposed solutions for improving the innovation to increase the sustainability potential.

Some facilities described the innovation as continuing to be used over time. This was characterized by thorough integration of the innovation into care routines. As one staff member explained,

“I really can’t remember like what our routine was before we started this”, and the musical cue “helps to cue us when we were supposed to do it because we didn’t…. always recognize that like 2 h has past; we have to go turn such and such, but you would hear the music, and you’re like, okay, we’ve got to go turn everybody. So, I think it kind of helped, but we liked the music too”.

In fact, for three of the eight facilities, the equipment was left in place as a result of staff requests to continue the program. As one staff participant said, “I liked it! I want it to continue, yeah! We still are implementing it”. By contrast, other groups described the innovation as not being used over time. One noted that “I think it was top down and decreased pressure ulcers obviously. I think overall it started well, but kind of slacked with all staff as the program went on probably due to lack of enforcement, on all of our parts”. Moreover, staff discussed a lack of consistency that eroded sustainability, “You would see some people one day do it, and the next day you wouldn’t see anybody do it; it’s like a consistency thing”.

The theme of brainstormed ideas was comprised of staff ideas or suggestions to improve the innovation and support adoption and sustainability. For example, the additional documentation of the innovation was identified as disadvantageous in our themes related to relative advantage. However, staff provided suggestions to fix this issue, such as developing a novel flag system to indicate whether someone was turned by changing the color of residents’ blankets or simplifying with a form of computer documentation. Furthermore, because the volume was not consistent throughout the facilities, staff suggested using wireless speakers with Wi-Fi boosters placed throughout the facility. These ideas or solutions for improving the innovation could potentially contribute to improved adoption and sustainability.

Overall, we identified both positive and negative examples of each attribute of the innovation (the relative advantage being the exception with only positive). Sixty-eight percent of all coded examples were coded as positive examples. Further, staff provided many suggestions for how to improve the innovation, most of which staff felt would improve the sustainability of the innovation.

## 4. Discussion

This study used Rogers’ [10] DOI model regarding innovation characteristics to guide the focus group interviews that explored staff perceptions of the attributes of the intervention that affected the decision to adopt, as well as staff perceptions regarding impact on PrUs. The staff were from eight LTC facilities that had completed a musical cueing intervention aimed at prompting staff to increase resident movement in order to reduce facility-acquired PrUs.

We learned that each innovation characteristic and sustainability was more complex than our team originally believed. Within each code, two themes emerged, reflecting either a positive or negative response to the characteristics of the innovation. We are the first, to our knowledge, to present the DOI characteristics with these two themes, because the characteristics have been previously considered as a degree of perception [10]. The largest portion of the focus groups’ discussion and coded quotes was related to the positively-toned comments and the perceived benefits, thereby demonstrating how meaningfully connected the innovation appeared to be with staff values and culture; the findings suggest a strong potential for adoption. In general, the staff described the musical cue as fun for all disciplines, joyous and helpful; it served as a reminder to increase residents’ movement. Staff considered the innovation to be better than the prior system for the implementation of standards of care about moving residents and described the music as improving the overall energy and mobility, both of which were perceived as improving quality of life; for example, dancing between staff and residents now occurs. The majority of staff generally perceived that the occurrence of PrUs was decreasing, and the musical cueing was seemingly simple to use and a good reminder to ensure staff consistency with resident movement. Overall, the participants in this study professed that there was an improvement using musical cues for PrU prevention over their previously used methods.

We recognize that a substantial portion (32%) of the discrete statements was related to utility issues. For example, the timing of the cue was periodically not ideal and subsequently affected non-nursing departments’ involvement; furthermore, there were facilities in which non-nursing disciplines did not participate during the entire implementation. However, the code for sustainability had a third theme “brainstormed ideas”, in which 5% of all statements focused on creative solutions for improving the innovation’s fit for future implementation, indicating that with minor adjustments or enhancements made, there was a perceived potential for the sustainability of the innovation. A number of participants explicitly expressed ideas aiming to simplify certain aspects in order to improve the intervention. Innovations that are perceived to be simple and easy to use and can be broken down into more manageable parts with few barriers to overcome will be assimilated and adopted more easily [19]. Our findings indicated that the musical cueing was perceived both as complex and, yet, as a seemingly simple innovation. The majority of concerns related to function was about the timing of the cues, sound quality of the music, the additional workload from the study-related documentation and the need for a strategy to increase other department involvement. All of these aspects are potentially modifiable to better fit each work environment.

This study adds to our understanding of the mechanisms by which an innovation’s characteristics yield (or fail to yield) the outcome of interest and might be successfully adopted in a particular context. For example, this study required additional documentation to be completed, which was considered to hinder adoption, but the additional study-related documentation would not be needed if the innovation were a permanent part of the facility system for PrU prevention. Another example would be how the music evolved and changed over time with new music requests every month. For some facilities, the music was made to be reflective of the residents’ era and genre choices, thereby enabling dancing in the halls and making both staff and residents happy. Each facility context is unique, making successful adoption for any length of time a complex process with a multiplicity of variables, which may or may not induce staff ambivalence and could negatively affect innovation adoption. Characteristics of the innovation are neither stable features nor sure determinants of the innovation’s adoption or assimilation, nor should they be. The ability to tailor aspects of an innovation (*i.e.*, music choice) to participant preferences appears to make it more appealing to the staff attitudes. No substantial differences in perceptions were found between the focus groups; the participant groups all described both positive and negative aspects of the innovation’s characteristics, which would influence their decision to either adopt or reject the intervention. Future research should focus on understanding how each innovation characteristic impacts outcomes of interest.

Staff largely perceived a positive impact of this innovation on residents’ movement and PrU outcomes. However, because PrU prevention is complex and context laden, clinicians need to pay close attention to those characteristics that facilitate or impede innovative implementation and guideline adoption and implementation. Research about implementing guidelines is needed to improve understanding about PrU prevention practices. In our study, all facilities that received the intervention experienced some reduction in facility-acquired PrUs by the end of study participation [8]. Our qualitative analysis leads us to believe that the impact of this intervention in part can be attributed to the perceived positive good fit of the intervention with the beliefs and values of the facility staff and residents. We postulate that the musical component of the innovation can influence the participants to connect and engage with the intervention and its aim of guideline implementation. 

A limitation of the study was that all facilities were from one corporation from the Midwestern U.S. that volunteered to participate in the study and may therefore be systematically different from facilities in other geographic regions or corporations, limiting generalizability. Furthermore, we used a convenience sample of staff that was available and willing to participate on the day of the scheduled focus group. Although it is possible that individuals who volunteered to participate did so because they favored the intervention, we believe that our efforts to recruit a diverse group of staff were effective, as reflected by 32% of the discussion consisting of negatively-toned comments. We used an unusual, semi-quantitative method for counting unique expressions of each subcategory and recognize that these counts may be open to bias. We were interested in exploring not only the qualitative differences in the innovation codes, but also how frequently they were expressed. To optimize confidentiality and coder blinding, participants were not named in coded transcripts, and no roles were stated, even though focus group leaders reported the overall focus group composition by gender and job title. Therefore, we were unable to compare the perceptions of staff in different roles. The focus groups only included one male, and thus, the findings might reflect gender bias. However, it is notable that a broad range of participant roles was represented. 

Tolba and Mourad [20] advanced the DOI model by proposing a “functional dimension” of the innovation, in which high complexity was considered to have a negative impact on innovation acceptance, and a “social dimension” of the innovation adoption that is influenced by different types of users and leaders [20]. Although these authors provided no definition of social and functional dimensions, our consideration of these dimensions enriched the interpretation of our findings. In our study, the social dimension embodied the organizational and cultural ways that people relate to an innovation, and the functional dimension embodied all of the activities and tasks that people recognize as relating to the way in which an innovation works or operates. These functional and social dimensions offer an expanded mechanism for explaining our focus group findings regarding perceptions of the innovation’s characteristics, outcomes and sustainability. The social dimension was evidenced in staff’s generally positively-toned perceptions regarding the innovation and its outcomes on resident movement and PrU prevention, suggesting that individual and cultural needs were being met and enhancing the potential for adoption of the innovation. The functional dimension was evidenced in the way staff perceived responsibilities and the utility of the innovation’s operational process, and this aspect of the conversation expressed more negatively-toned comments than did the social dimension.

Future research on innovations will depend upon a better understanding of their social dimensions, because it is the interaction among the innovation, the intended adopter(s) and a particular context that determines whether adoption of the innovation is successful and is sustainable [19]. Furthermore, we would suggest that future research address whether improved outcomes are contingent on an intervention assimilation period. This pressure ulcer prevention intervention study’s results showed that the frequency of new facility-acquired PrUs per assessment was less than 2% within three months of intervention deployment in both facility groups and below 1% during the final three months of the study [8]. The benefits of this particular innovation might extend beyond the intended mobility-related benefits of this simple, inexpensive intervention to the benefits afforded by sensory stimulation. Both staff and residents expressed satisfaction with the music. Music from the residents’ era was chosen by staff in some facilities, because it brought a great deal of pleasure to residents, prompting memories and the sharing of stories, which, in turn, improved staff morale [8].

## 5. Conclusions

We conclude that musical cueing for the promotion of the consistent implementation of guidelines regarding LTC resident movement aimed at PrU prevention is worth further exploration. This intervention had valuable unanticipated consequences, such as improving social connections by increasing communication frequency and quality between staff and residents. The intervention appears to enhance the social aspects of the care environment, which might increase innovation uptake and adoption among the LTC staff. Multiple departments participated initially, but there were some unanticipated consequences that interfered with the successful participation of these disciplines over time. However, adoption is a process rather than an event, with different concerns being dominant at different points in the process; the decision to adopt an innovation is also based on an individual’s perception of the innovation’s worth relative to other ways of accomplishing the same goal. Moreover, what one staff member perceives as easy and valuable might be considered unimportant, unpleasant or even hard for another staff member. The impact of musical cueing on other guideline implementation and clinical outcomes requires additional validation and, thus, merits future research.
